# Identification of U937^JAK3-M511I^ Acute Myeloid Leukemia Cells as a Sensitive Model to JAK3 Inhibitor

**DOI:** 10.3389/fonc.2021.807200

**Published:** 2022-01-17

**Authors:** Hongfei Si, Jie Wang, Rui He, Xiuwen Yu, Shan Li, Jing Huang, Jie Li, Xia Tang, Xiaojuan Song, Zhengchao Tu, Zhang Zhang, Ke Ding

**Affiliations:** ^1^ International Cooperative Laboratory of Traditional Chinese Medicine Modernization and Innovative Drug Development of Chinese Ministry of Education (MOE), Guangzhou City Key Laboratory of Precision Chemical Drug Development, School of Pharmacy, Jinan University, Guangzhou, China; ^2^ Guangzhou Institutes of Biomedicine and Health, Chinese Academy of Sciences, Guangzhou, China

**Keywords:** JAK3 mutation, U937, AML, JAK3 selective inhibition model, JAK3 inhibitor

## Abstract

Mutated JAK3 has been considered a promising target for cancer therapy. Activating mutations of JAK3 are observed in 3.9%–10% of acute myeloid leukemia (AML) patients, but it is unclear whether AML cells are sensitive to JAK3 inhibitors, and no disease-related human AML cell model has been reported. We have identified U937 as the first human AML cell line expressing the JAK3^M511I^ activated mutation and confirmed that JAK3 inhibitors sensitively suppress the proliferation of U937 AML cells.

## Introduction

JAK3 belongs to the Janus non-receptor tyrosine kinase (JAK) family. JAK3 is different from the other members of the family including JAK1, JAK2, and Tyk2. It is exclusively expressed in the hematopoietic compartment and interacts with the common γ chain of several interleukin receptors where it mediates the development and functions of T cells, B cells, and natural killer (NK) cells. Activated mutations of JAK3 have been found in a variety of hematopoietic malignancies, including acute myeloid leukemia (AML), T-cell acute lymphoblastic leukemia (T-ALL), and T-cell prolymphocytic leukemia (T-PLL) (1–3). For instance, JAK3 mutations account for 3.9%–10% of AML patients (4, 5). Approximately 10%–16% of T-ALL patients carry at least one type of JAK3 mutation, and JAK3^M511I^ is the most frequently detected mutation (6).

Further investigations confirmed that several JAK3 mutations lead to cytokine independent proliferation and malignant tumor transformation in Ba/F3 cells or in animals (1, 7–9). These data collectively supported a hypothesis that mutated JAK3 could be considered a promising target for cancer therapy. However, it was unclear whether AML cells are sensitive to JAK3 inhibitors, and no disease-related human AML cell model has been reported.

Herein, we report the identification of U937 acute monocytic leukemia cells that harbor the JAK3^M511I^ mutation and are sensitive to selective, small-molecule JAK3 inhibitors. This cell line could serve as a model for the biological investigation of mutated JAK3 kinases and for selective screening of JAK3 inhibitors.

## Methods

### Agents

Upadacitinib, filgotinib, fedratinib, ruxolitinib, baricitinib, tofacitinib, and peficitinib were purchased from the Topscience Company (Shanghai, China). PF-06651600 was purchased from the Selleckchem Company (Houston, TX, USA). These compounds were dissolved in dimethyl sulfoxide (DMSO) (10 mM) and stored at −20°C. Primary antibodies against JAK1 (29261S), phosphor-JAK1 (74129S), JAK2 (3230S), phosphor-JAK2 (3776S), JAK3 (8827S), phosphor-JAK3 (5031S), STAT3 (9139S), phosphor-STAT3 (9145S), STAT5 (94205S), phosphor-STAT5 (9356S), CDK4 (12790S), CDK6 (13331S), Cyclin B1 (12231S), Cyclin D3 (2936S), Cyclin E1 (20808S), PARP (9532S), GAPDH (2118S), and secondary antibodies were purchased from Cell Signaling Technology (Boston, MA, USA).

### Cell Culture

All cell lines were purchased from the Cell Resources Center, Shanghai Academy of Life Sciences, Chinese Academy of Sciences. U937, OCI-LY3, MOLT-4, Jurkat, and Daudi cells were grown in RPMI-1640 (Biological Industries); MV4-11 was grown in IMDM (SIGMA) supplemented with 10% fetal bovine serum (FBS) (Biological Industries) and 1% penicillin-streptomycin (Biological Industries). All cells were grown at 37°C in a humidified 5% CO_2_ atmosphere.

### *In Vitro* Kinase Assays

JAK1, JAK2, JAK3, and a Z’-Lyte kinase assay kit were purchased from Invitrogen (Waltham, MA, USA), and the assays were performed according to the manufacturer’s instructions.

### Construction of Ba/F3-JAK Stable Cells

Murine Ba/F3 cells were purchased from DSMZ (Deutsche Sammlung von Mikroorganismen und Zellkulturen). Ba/F3 cell lines stably expressing JAK1 and JAK2 were self-established through electroporation of pCDNA3.1-TEL plasmids. JAK3 (M511I) was produced from pCDNA3.1 plasmids using the Amaxa Cell Line Nucleofector Kit V (Lonza, Cologne, Germany). Stable cell lines were selected by G418 (Merck, NJ, USA) and subsequent withdrawal of IL-3 (R&D, MN, USA). All Ba/F3 stable cell lines were confirmed by positive drug tests, DNA sequencing, and Western blotting (WB) analysis. Parental Ba/F3 cells were maintained in RPMI-1640 supplemented with 10% FBS (Biological Industries) and 1% penicillin-streptomycin (Biological Industries) and 10 ng/ml of IL-3, while all Ba/F3 stable cell lines were cultured in the same medium without IL-3.

### Anti-Proliferation Activity

Cells in the logarithmic phase were seeded in 96-well plates at 8,000–10,000 cells/well. Twenty-four hours after seeding, cells were treated with serial dilutions of test compounds at a final concentration of 0.1% in DMSO in medium and incubated for an additional 72 h. At the end of treatment, cell counting kit-8 (CCK-8; Dojindo Laboratories, Kumamoto, Japan) was added into the 96-well plates (10 μl/well) and incubated with the cells for 1–3 h. OD_450_ and OD_650_ were determined with a microplate reader. The absorbance value (A) for each well was calculated as OD_450_ − OD_650_. The cell viability rate for each well was calculated as V% = (A_s_ − A_c_)(A_b_ − A_c_) × 100%. The data presented are the mean values from at least three independent experiments. IC_50_ values were calculated by log(inhibitor) vs. response-Variable slope (four parameters) using GraphPad Prism 8.0 software (GraphPad Software, Inc.). A_s_ represents the absorbance value of the test compound well, A_c_ is the absorbance value of the well with neither cells nor test compounds, and A_b_ is the absorbance value of the well with cells and vehicle control.

### RNA Interference

Synthetic JAK3 small interfering RNA (siRNA) and its negative control (NC) were purchased from RiboBio (Guangzhou, China). Specific siRNAs targeting JAK3 are as follows: si-JAK3: 5′-GCAGACACTTAGCTTGGAA-3′. Transfection of 2 × 10^6^ U937 and OCI-LY3 cells with 9 pmol of JAK3 siRNA was achieved by electroporation, following the manufacturer’s procedures given in the Cell Line Nucleofector™ Kit V from Lonza (Cologne, Germany). After 48 h, cells were harvested using sodium dodecyl sulfate (SDS) lysis buffer, and then proteins were analyzed using WB.

### Quantitative Real-Time PCR

qPCR was conducted to quantify the expression level of JAK3 mRNA. Total RNA in U937, MOLT-4, Jurkat, Daudi, OCI-LY3, and MV4-11 cells was extracted by TRIzol (Invitrogen, CA, USA) according to the manufacturer’s instructions. RNA was then reverse transcribed into cDNA with PrimeScript™ RT reagent Kit (TaKaRa, Tokyo, Japan). Sequentially, qPCR system was prepared following the manufacturer’s instructions in the TB Green^®^ Premix Ex Taq™ (TaKaRa) using 200 ng of cDNA. The primers applied in this current study are as follows:

JAK3-Forward: 5′-CCGTCATTCGTGACCTCAAT-3′
JAK3-Reverse: 5′-CATAGAGCTGGGCACCATTC-3′
GAPDH-Forward: 5′-GGCTCTCCAGAACATCATCCCTGC-3′
GAPDH-Reverse: 5′-GGGTGTCGCTGTTGAAGTCAGAGG-3′

Finally, each sample was analyzed in triplicate using a CFX 96 Thermocycler (Bio-Rad, USA). Relative quantification of genes was analyzed in accordance with the 2^−ΔΔCt^ method (ΔΔCt = ΔCt [target] − ΔCt [control]). GAPDH was used as an endogenous control.

### cDNA Sequencing

Total RNA in the cells was extracted using the TRIzol reagent according to the manufacturer’s instructions. The RNA was then reverse transcribed into cDNA with PrimeScript™ II 1st Strand cDNA Synthesis Kit (TaKaRa, Tokyo, Japan). PCR amplification of cDNA and sequencing were performed using JAK3 primers

Forward: 5′-CCTTCGAAAGTCCAGGGTC-3′
Reverse: 5′-AAGGACAGGGAGTGGTGTTTG-3′

Sequence analysis of bidirectional sequence traces was performed using DNA Star (Madison, WI) and Mutation Surveyor (State College, PA). Bidirectional sequence traces were assembled and analyzed for mutations using the Mutation Surveyor, version 2.5 (SoftGenetics, State College, PA).

### Cell Cycle Analysis

Cells were treated with indicated concentrations of PF-06651600 or DMSO for 24 h. About 6 × 10^5^ cells were resuspended in 150 μl of BD Cytofix/Cytoperm buffer solution I for 10 min at room temperature (rt). The cell suspensions were added with BD Cytofix/Cytoperm buffer solution II and incubated with another 10 min at rt and then incubated with 200 μl of propidium iodide buffer (0.1 mg/ml) in the dark for 10 min at 4°C. Assays were performed with a Guava easyCyte flow cytometer (Merck, USA).

### Apoptosis Assay

Cells were treated with indicated concentrations of PF-06651600 or DMSO for 48 h at 37°C. After incubation, cells were collected and washed twice with pre‐cold phosphate-buffered saline (PBS). About 6 × 10^5^ cells were resuspended in 100 μl of 1× BD binding buffer solution (#556454, BD) and then stained with 7‐ADD (#559925, BD) and Annexin V‐PE (#556422, BD) in the dark for 15 min. Finally, 400 μl of 1× BD binding buffer solution was added to stop the dyeing. The cells were then measured on a Guava easyCyte flow cytometer (Merck, USA).

### Western Blotting Analysis

Cells at a cell density of 8 × 10^5^ cells/ml were treated with different concentrations of compounds or DMSO for the indicated time. The cells were then lysed with 1× SDS sample lysis buffer, as recommended by CST. After ultra‐sonication and boiling, the cell lysates were separated by SDS–polyacrylamide gel electrophoresis (SDS‐PAGE) and then transferred to a polyvinylidene difluoride (PVDF) membrane (Millipore). After being blocked with 5% bovine serum albumin (BSA) in tris-buffered saline and polysorbate (TBST) solution (0.5% Tween‐20) at rt for 4 h, the membranes were incubated with the corresponding primary antibody (1:2,000–1:200) overnight at 4°C. After washing 3–5 times with TBST, the horseradish peroxidase (HRP)‐conjugated secondary antibodies were incubated for 2 h. The protein signals were detected with Amersham Imager 600 system (GE, Boston, MA, USA) using an ECL Western blotting detection kit (Thermo Fisher Scientific, Waltham, MA, USA).

## Results

### JAK3 Inhibitors Selectively Suppress the Proliferation of U937 Acute Myeloid Leukemia Cells

In order to investigate the effects of JAK3 inhibitors on leukemia cells, we selected the approved JAK inhibitors and a panel of hematological malignancy cells. Firstly, we used the Z’-Lyte kinase assay to validate the kinase inhibitory activities and isoform selectivity of a panel of Food and Drug Administration (FDA)-approved or clinically investigated JAK inhibitors including upadacitinib, filgotinib, fedratinib, ruxolitinib, baricitinib, PF-06651600 (ritlecitinib), tofacitinib, and peficitinib. It was confirmed that only PF-06651600 demonstrated selective JAK3 inhibition with an IC_50_ value of 0.0012 ± 0.00047 μM, while its potency against JAK1 or JAK2 was approximately 3,000- or 5,700-fold less, respectively (**Table 1**).

**Table 1:** Kinase assay and anti-proliferative activities of JAK3 inhibitors.

IC_50_ (μM)	Target	Kinase			Cell line						
		JAK1	JAK2	JAK3	U937	MOLT-4	OCI-LY3	Jurkat	Daudi	HL-60	MV4-11
Upadacitinib	JAK1	0.0021 ± 0.00035	0.0055 ± 0.0012	0.030 ± 0.0085	0.25 ± 0.03	>10	>10	>10	>10	>10	>10
Filgotinib	JAK1	0.40 ± 0.11	1.75 ± 0.29	>10	>10	>10	>10	>10	>10	>10	>10
Fedratinib	JAK2	0.17 ± 0.0027	0.029 ± 0.0061	1.69 ± 0.92	2.53 ± 0.17	2.72 ± 0.54	3.18 ± 0.13	1.42 ± 0.32	3.92 ± 0.21	2.44 ± 0.16	>10
Ruxolitinib	JAK1/2	0.0053 ± 0.00042	0.0030 ± 0.00064	0.086 ± 0.042	0.70 ± 0.11	>10	>10	>10	>10	>10	>10
Baricitinib	JAK1/2	0.0082 ± 0.0011	0.0086 ± 0.0023	0.35 ± 0.12	3.81 ± 1.20	>10	>10	>10	>10	>10	>10
PF-06651600	JAK3	3.63 ± 0.47	6.89 ± 0.95	0.0012 ± 0.00047	0.027 ± 0.004	>10	>10	>10	>10	>10	>10
Tofacitinib	Pan-JAKs	0.015 ± 0.0029	0.034 ± 0.0096	0.015 ± 0.0061	0.18 ± 0.04	>10	>10	>10	>10	>10	>10
Peficitinib	Pan-JAKs	0.0097 ± 0.0024	0.029 ± 0.0076	0.0093 ± 0.0037	0.09 ± 0.02	2.22 ± 0.08	3.42 ± 0.01	2.32 ± 0.82	5.82 ± 0.15	4.93 ± 1.22	9.19 ± 0.98

Kinase inhibitory was performed by Z’-Lyte method-based fluorescence resonance energy transfer (FRET) principle. The anti-proliferative activities of the compounds were evaluated using cell counting kit-8 (CCK-8) assay, which indicated that cells were treated with compounds or 0.1% dimethyl sulfoxide (DMSO) for 72 h. Data are expressed as mean ± SD of at least three independent experiments.

We next evaluated the antiproliferative activity of the JAK inhibitors against a panel of hematological malignancy cells including U937, MOLT-4, OCI-LY3, Jurkat, Daudi, HL-60, and MV4-11. It was found that all the cell lines except U937 were almost totally inactive with respect to the inhibitors regardless of their isoform selectivity. Interestingly, the JAK inhibitors demonstrated differentiated antiproliferative activity against U937 AML cells, and this was highly correlated with their JAK3 inhibitory potencies. For instance, a selective JAK3 inhibitor exhibited the best kinase inhibitory potency with an IC_50_ value of 1.2 nM and also displayed the strongest antiproliferative activity against U937 cells with an IC_50_ value of 27 nM. The pan JAK inhibitors upadacitinib, tofacitinib, and peficitinib also exhibit strong JAK3 inhibition with IC_50_ values of 30, 15, and 9.3 nM, respectively, and their IC_50_ values against U937 cells are 250, 120, and 90 nM, respectively. The selective JAK1 inhibitor filgotinib did not obviously suppress JAK3 kinase activity and was also almost totally inactive in the U937 cell growth inhibition assay. Fedratinib and baricitinib weakly suppressed the activity of JAK3, and they also demonstrated micromolar IC_50_ values in inhibition of the proliferation of U937 cells. Ruxolitinib demonstrated sub-micromole antiproliferative activity against U937 cells because of its reasonable inhibition of JAK3 (**Table 1**).

### JAK3 Overexpression and Mutant Activation in U937 Cells

**Figure 1:**
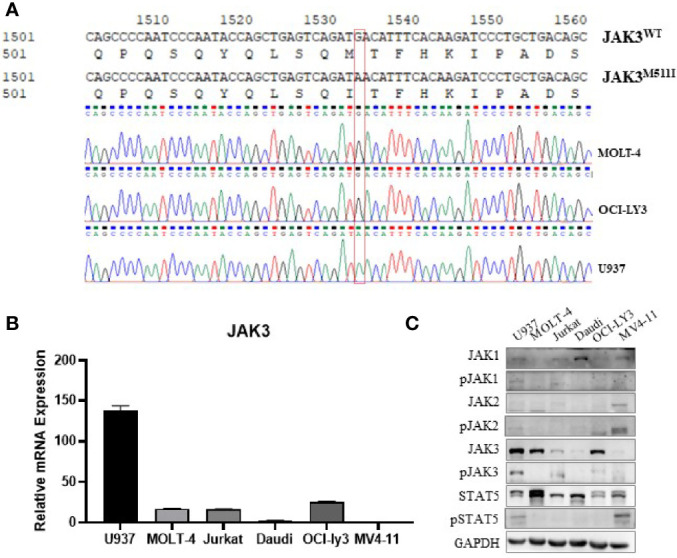
JAK3 is M511I mutant activation in U937 cells. **(A)** Sanger sequencing electropherogram of the JAK3 cDNA of U937, MOLT-4, and OCI-LY3 cells. **(B)** Relative JAK3 mRNA expression levels in U937, MOLT-4, Jurkat, Daudi, OCI-LY3, and MV4-11 cell lines. **(C)** The activation of JAK3-STAT3/5 signal pathway between different cell lines (U937, MOLT-4, Jurkat, Daudi, OCI-LY3, and MV4-11) was compared by Western blotting.

The U937 cell line was originally established from the pleural effusion of a patient with histiocytic lymphoma and is usually considered to be an AML cell line (10, 11). To get some insight into the reason why the U937 cell line is sensitive to JAK3 inhibitors, we amplified and sequenced the JAK3 complementary DNA (cDNA) in U937, MOLT-4, OCI-LY3, Jurkat, Daudi, HL-60, and MV4-11 cells. The Sanger sequencing electropherogram results revealed that U937 cells harbored the JAK3 p.M511I mutation, which is absent in the other cells (**Figure 1A**).

We also compared the JAK3 mRNA expression level and activation of the JAK3 signaling pathway in the aforementioned cell lines. It was shown that U937 cells demonstrated the highest levels of mRNA expression of JAK3 (**Figure 1B**), JAK3 protein, phosphorylated JAK3 (p-JAK3), and downstream phosphorylated STAT5 (p-STAT5), suggesting signaling by the constitutively activated JAK3 (**Figure 1C**). Although MOLT4 and OCI-LY3 cell lines also expressed high levels of wild-type JAK3, the downstream STAT5 activation was not detected.

### JAK3-M511I Mutation Malignant Transformed Ba/F3 Cells

In order to confirm that the selective anti-proliferative activity of the JAK inhibitors is mediated by JAK3^M511I^ mutated kinase inhibition, Ba/F3 cell models stably expressing TEL-JAK1, TEL-JAK2, and JAK3^M511I^ were established (**Figure S1**). It was validated that the JAK3^M511I^ mutation induced cytokine-independent proliferation of Ba/F3 cell lines. A CCK-8 assay showed that the selective JAK3 inhibitor PF-06651600 strongly inhibits the Ba/F3-JAK3-M511I cells with an IC_50_ value of 0.030 ± 0.01 μM, but the other JAK inhibitors were clearly less potent (**Table 2**). These results further confirmed that JAK3-M511I mutation mediates the sensitivity of JAK3 to its inhibitors.

**Table 2:** JAK3 inhibitors selectively suppress the proliferation of Ba/F3-JAK3-M511I cells.

IC_50_ (μM)	Upadacitinib	Filgotinib	Fedratinib	Ruxolitinib	Baricitinib	PF-06651600	Tofacitinib	Peficitinib
Ba/F3-TEL-JAK1	0.075 ± 0.008	>10	1.96 ± 0.24	0.25 ± 0.02	0.71 ± 0.10	>10	0.76 ± 0.08	0.60 ± 0.08
Ba/F3-TEL-JAK2	0.18 ± 0.01	>10	1.83 ± 0.06	0.10 ± 0.008	0.37 ± 0.05	>10	1.20 ± 0.04	1.29 ± 0.09
Ba/F3-JAK3 (M511I)	0.098 ± 0.047	7.19 ± 2.5	2.84 ± 0.39	0.11 ± 0.01	0.36 ± 0.01	0.030 ± 0.01	0.023 ± 0.006	0.10 ± 0.05
Ba/F3	0.90 ± 0.12	>10	3.47 ± 0.33	0.42 ± 0.04	1.33 ± 0.15	>10	3.37 ± 0.03	1.56 ± 0.09

The antiproliferative activities of the compounds were evaluated using cell counting kit-8 (CCK-8) assay. The cells were treated with compound or 0.1% dimethyl sulfoxide (DMSO) for 72 h. Data are expressed as mean ± SD of at least three independent experiments.

### The Growth of U937 Cells Is Dependent on JAK3 Protein

**Figure 2:**
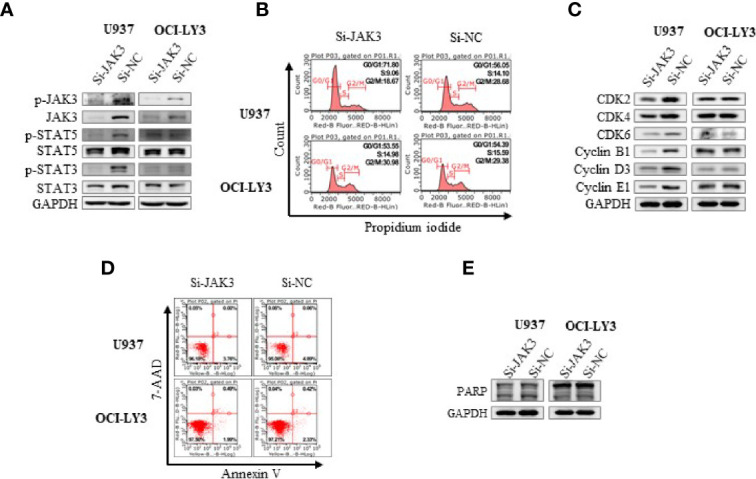
Si-JAK3 suppressed activation of JAK3-STAT3/5 signal pathway and induced G0/G1 phase arrest in U937 cells. **(A)** Western blotting analysis of whole-cell lysates after electrotransformation of JAK3 siRNA for 72 h, and the JAK3-STAT3/5 signal pathway in U937 and OCI-LY3 cells. The change of cell cycle **(B)**, cycle-related proteins **(C)**, cell apoptosis **(D)**, and apoptosis-related protein **(E)** was analyzed by flow cytometry or Western blotting.

In order to further validate the contribution of JAK3^M511I^ to the downstream signal activation in U937 cells, the mutated kinase was selectively knocked down with si-RNA. It was shown that activation of the JAK3 downstream signals was significantly blocked as evidenced by decreasing levels of phosphorylated STAT3 and STAT5 (**Figure 2A**). Selective knockdown of JAK3^M511I^ obviously induced G0/G1 phase cell cycle arrest (**Figure 2B**) and significantly decreased the expression of cell cycle-related proteins including CDK2, CDK4, CDK6, Cyclin B1, Cyclin D3, and Cyclin E1 (**Figure 2C**), but JAK3 knockdown did not cause obvious apoptosis of U937 cells (**Figures 2D, E**). In a parallel comparison, JAK3 knockdown was found not to affect the downstream signals or induce cell cycle arrest in OCI-LY3 cells with wild-type JAK3.

### JAK3 Inhibitor Inhibits JAK3 Signal Pathway and Induces G0/G1 Phase Arrest But Fails to Cause Apoptosis in U937 Acute Myeloid Leukemia Cells

**Figure 3:**
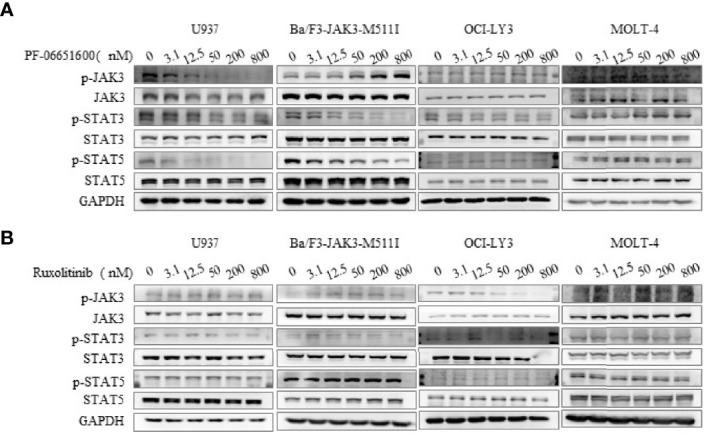
Selective JAK3 inhibitor inhibited the activation of JAK3 and downstream signaling molecules in U937 cells. The effects of PF-06651600 **(A)** or ruxolitinib **(B)** on JAK3-STAT3/5 signal pathway in U937, Ba/F3-JAK3-M511I, OCI-LY3, and MOLT-4 cancer cell lines.

**Figure 4:**
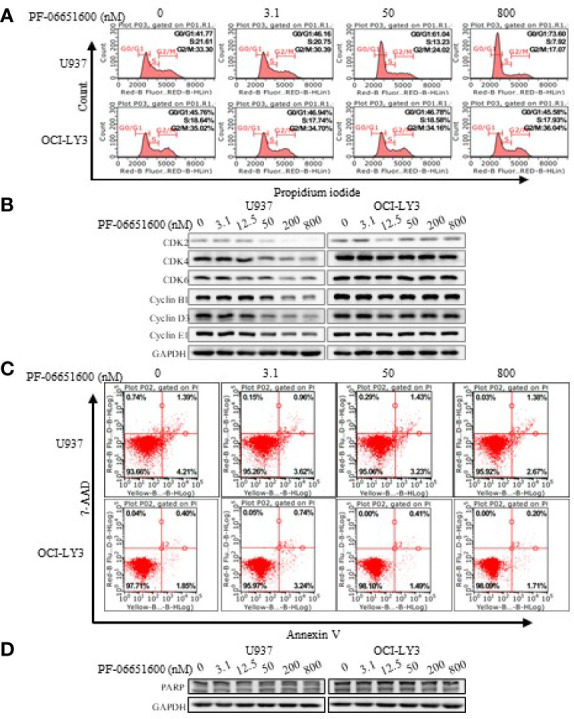
Selective JAK3 inhibitor induced G0/G1 phase arrest in U937 cells. The change of cell cycle **(A)** and cycle-related proteins **(B)** was analyzed by flow cytometry and Western blotting 24 h after PF-06651600 treatment. Cell apoptosis **(C)** and apoptosis-related protein **(D)** were analyzed by flow cytometry and Western blotting 24 h after PF-06651600 treatment.

To further validate the effects of JAK3 inhibitors on U937 cells, we performed the signal pathway and cell flow cytometry analyses. The WB results displayed that selective JAK3 inhibitor PF-06651600 potently suppressed the activation of JAK3 and the downstream signals in a dose-dependent manner in U937 cells (**Figures 3A, B**). However, in the Ba/F3-JAK3 M511I cell line, although PF-06651600 inhibited the downstream signaling factors of JAK3, it enhanced the phosphorylation of JAK3 (**Figures 3A, B**). Incubation with the inhibitor also selectively caused U937 cells to undergo dose-dependent G0/G1 phase cell cycle arrest, while inducing apoptosis only barely (**Figure 4**). Treatment with PF-06651600 did not obviously affect the cell fate or the phenotypes of OCI-LY3 cells (**Figure 4**). Collectively, these results suggest that JAK3^M511I^ mutation is indeed a major cause of the downstream signal activation and contributes to cell proliferation in U937 cells.

## Discussion

Despite the progress that has been made in the treatment of AML, many challenges remain in the targeting of drug resistance and high-risk subtypes, and the long-term patient survival is still unacceptably low. The important role of JAK3 mutations in leukemia has become increasingly clear in recent years. The frequency of JAK3 mutations in AML is about 3%–10%, and JAK3 mutations are also common in other blood cell tumors such as T-ALL and T-PLL (1–3, 7). There are currently no FDA-approved drugs for JAK3 mutation subtypes. However, previous studies were mainly based on artificially constructed tool cells and did not have access to disease-related human cell line models.

A panel of hematological malignancy cells was screened with an anti-proliferation assay for JAK inhibitors. We found that the U937 cell line is sensitive to JAK3 inhibitors, with high expression of JAK3 and obvious activation of its downstream pathway. Previous studies had shown that the monocytic cell line U937 can express JAK3 (12), and cDNA sequencing analysis confirmed that the U937 cell line harbors a homozygous JAK3^M511I^ mutation. Further studies have shown that both homozygous mutations and heterozygous mutations in JAK3 have been found in clinical samples, and homozygous mutations have a stronger transformation ability and a poorer prognosis (6, 8).

Ba/F3 transformation showed that JAK3^M511I^ mutation could induce Ba/F3 non-interleukin-dependent proliferation and sensitivity to JAK3 inhibitors. This transformation ability of JAK3^M511I^ and sensitivity to JAK3 inhibitors are consistent with previous studies (9, 13). Wild-type JAK1 and JAK2 require mediated activation by interleukin receptors and cannot induce Ba/F3 non-interleukin-dependent proliferation. Therefore, the Ba/F3 model we constructed (JAK1 and JAK2) is expressed by TEL fusion, which also exists in clinical samples (14). The anti-proliferation test showed that consistent with previous studies (15), ruxolitinib inhibits both fusion mutations.

In U937 cell line, knockdown or inhibition of JAK3 or JAK3 kinase activity significantly inhibits downstream pathways and induces G0/G1 phase cell cycle arrest, but has no effect on apoptosis. The U937 cell line is highly dependent on JAK3 activity. Some studies have shown that inhibition of JAK3 kinase activity can induce apoptosis, but the inhibitor used in these studies was tofacitinib, a pan-JAK inhibitor, so it may be an off-target activity that induces apoptosis, or it may be caused by the defects in the artificial construction tools used in the study (8, 13, 16). In the Ba/F3-JAK3-M511I cell line, although PF-06651600 inhibited the downstream signaling factors of JAK3, it enhanced the phosphorylation of JAK3. It has been reported that activation of JAK pathway upregulates the expression of SOCS protein in Ba/F3-JAKs cell line, and SOCS directly inhibits the phosphorylation of JAKs, playing a feedback regulation role (17). Inhibition of the JAK-STAT pathway resulted in decreased SOCS expression and increased JAK phosphorylation. Since the inhibitor blocked the JAK-STAT pathway, increased JAK phosphorylation did not promote cell proliferation and survival (17). The upregulation of JAK3 activation may be related to the negative feedback mediated by SOCS1 protein, which is highly expressed in Ba/F3 cells but not in U937 cells (18).

In summary, we identified U937 AML cells as the first human leukemia cell line expressing the JAK3^M511I^ mutation. This might provide a valuable cell model for JAK3 inhibitor screening and assist in an understanding of its involvement in human diseases.

## Data Availability Statement

The raw data supporting the conclusions of this article will be made available by the authors, without undue reservation.

## Author Contributions

HS, JW, and RH: design of methodology. XY and XT: creation of models. XS and ZT: kinase assays. ZZ and KD: oversight and leadership responsibility for the research activity planning and execution. All authors contributed to the article and approved the submitted version.

## Funding

This study was funded by the International Science and Technology Innovation Cooperation Program of the State Key Research and Development Program (SQ2019YFE010401); General Project of National Natural Science Foundation of China (81973158); Guangdong Province Key Research and Development Field (2019B020204002); General Project of the Natural Science Foundation of Guangdong Province (2019A1515011235); and Guangzhou Basic and Applied Basic Research Project (202002030414).

## Conflict of Interest

The authors declare that the research was conducted in the absence of any commercial or financial relationships that could be construed as a potential conflict of interest.

## Publisher’s Note

All claims expressed in this article are solely those of the authors and do not necessarily represent those of their affiliated organizations, or those of the publisher, the editors and the reviewers. Any product that may be evaluated in this article, or claim that may be made by its manufacturer, is not guaranteed or endorsed by the publisher.

## Supplementary Material

The Supplementary Material for this article can be found online at: https://www.frontiersin.org/articles/10.3389/fonc.2021.807200/full#supplementary-material
