# Supplementary material for: Identification of U937JAK3-M511I Acute Myeloid Leukemia Cells as a Sensitive Model to JAK3 Inhibitor
Source: Front Oncol. 2022 Jan 17;11:807200. doi: 10.3389/fonc.2021.807200 (PMC8802890; doi:10.3389/fonc.2021.807200)
Supplement: Supplementary file 1 [file Image_1.pdf]

Identification of U937$^{JAK3\text{-}M511I}$ AML Cells as a sensitive model to JAK3 inhibitor

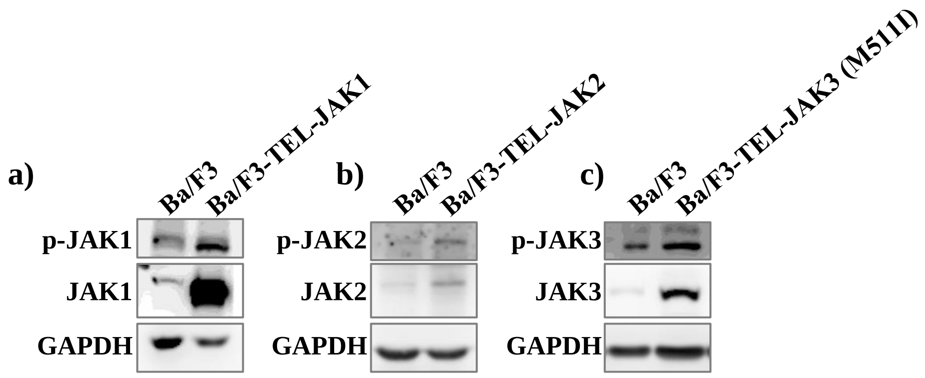

Fig S1. Identification of Ba/F3 stable cell lines.
The Ba/F3-TEL-JAK1 (a), Ba/F3-TEL-JAK2 (b) and Ba/F3-JAK3 (M511I) (c) stable cell lines were analyzed by western blot.
